# Clinician Knowledge and Beliefs after Statewide Program to Promote Appropriate Antimicrobial Drug Use

**DOI:** 10.3201/eid1106.050144

**Published:** 2005-06

**Authors:** Karen M. Kiang, Burney A. Kieke, Kathryn Como-Sabetti, Ruth Lynfield, Richard E. Besser, Edward A. Belongia

**Affiliations:** * Minnesota Department of Health, Minneapolis, Minnesota, USA;; † Centers for Disease Control and Prevention, Atlanta, Georgia, USA;; ‡ Marshfield Clinic Research Foundation, Marshfield, Wisconsin, USA

**Keywords:** Antibiotic resistance, reducing antimicrobial resistance, Drug resistance

## Abstract

In 1999, Wisconsin initiated an educational campaign for primary care clinicians and the public to promote judicious antimicrobial drug use. We evaluated its impact on clinician knowledge and beliefs; Minnesota served as a control state. Results of pre- (1999) and post- (2002) campaign questionnaires indicated that Wisconsin clinicians perceived a significant decline in the proportion of patients requesting antimicrobial drugs (50% in 1999 to 30% in 2002; p<0.001) and in antimicrobial drug requests from parents for children (25% in 1999 to 20% in 2002; p = 0.004). Wisconsin clinicians were less influenced by nonpredictive clinical findings (purulent nasal discharge [p = 0.044], productive cough [p = 0.010]) in terms of antimicrobial drug prescribing. In 2002, clinicians from both states were less likely to recommend antimicrobial agent treatment for the adult case scenarios of viral respiratory illness. For the comparable pediatric case scenarios, only Wisconsin clinicians improved significantly from 1999 to 2002. Although clinicians in both states improved on several survey responses, greater overall improvement occurred in Wisconsin.

In the United States, a substantial proportion of antimicrobial agents are prescribed for acute respiratory infections, including colds, upper respiratory infections (URIs), acute bronchitis, pharyngitis, sinusitis, and otitis media (1–9). Many of these illnesses are viral, and antimicrobial agents offer no benefit. However, widespread and inappropriate use of antimicrobial agents for viral illnesses has contributed to the emergence of infections caused by antimicrobial drug-resistant organisms such as *Streptococcus pneumoniae* (10–12). The proportion of invasive infections caused by penicillin-nonsusceptible *S*. *pneumoniae* increased nationally from 1% in 1992 to 27% in 2000 (10). Multidrug resistance has also occurred with increasing frequency: the proportion of *S*. *pneumoniae* isolates nonsusceptible to ≥3 classes of antimicrobial drugs increased from 7% in 1995 to 19% in 2000 (10). Multiple studies have shown a strong and consistent association between recent antimicrobial drug use and infection with a drug-resistant strain of pneumococcus (13–18). More recently, rapidly increasing rates of fluoroquinolone use have also been implicated in the emergence of fluoroquinolone-resistant pneumococcal infections (19–22). The increase in antimicrobial drug–resistant infections has economic as well as clinical implications; the annual cost of unnecessary antimicrobial drug prescribing for acute respiratory infections has been estimated to be ≈$726 million (5).

Throughout the previous decade, multiple interventions aimed at patients and clinicians have been implemented to promote appropriate antimicrobial drug use and prevent the development of antimicrobial resistance. In Wisconsin, a multifaceted educational campaign focusing on clinicians and the public was launched in late 1999 by the Wisconsin Antibiotic Resistance Network (WARN). Clinician education included presentations at professional meetings, conferences, and grand rounds; continuing medical education satellite conferences; distribution of slide presentations on CD-ROM; and multiple mailings of educational materials to all primary care clinicians. The public education component consisted of multilingual brochures and posters, tear-off sheets, coloring sheets, stickers, magnets, and handouts. These items were distributed statewide to clinics, managed care organizations, pharmacies, childcare facilities, and community groups. Mass media activities included radio advertisements statewide and paid television advertisements in selected markets. A more detailed account of WARN campaign activities is provided in the accompanying article (23). The purpose of this study was to assess the impact of the WARN campaign on the knowledge, beliefs, and decision-making of Wisconsin primary care clinicians regarding appropriate antimicrobial drug use for upper respiratory infections.

## Methods

### Design and Study Population

The study consisted of serial cross-sectional surveys in 2 states with pre- and postintervention measurements. Minnesota served as a control state to distinguish intervention-related changes from the regional secular trend. Minnesota was selected for geographic proximity and similarity in terms of population size and racial/ethnic distribution. Before 2002, educational activities on appropriate antimicrobial drug use were limited in Minnesota. Approval for this study was obtained from the institutional review board of CDC.

Eligible participants for the survey included physicians, nurse practitioners, and physician assistants. Practice specialties for physicians and physician assistants included family practice, pediatrics, internal medicine, emergency medicine, and general practice. Specialties for nurse practitioners included family practice and pediatrics. In 1999 and 2002, independent random samples were selected from Wisconsin and Minnesota licensing databases. The 1999 sampling frame was 7,113 in Wisconsin and 6,335 in Minnesota; the 2002 sampling frame was 6,218 in Wisconsin and 5,800 in Minnesota. The survey sample included 400 Wisconsin and 400 Minnesota clinicians in 1999, and 600 Wisconsin and 400 Minnesota clinicians in 2002. The baseline sample size was selected to provide >80% power to detect a 15% increase in the proportion of clinicians giving the correct or desired response to a specific survey question (α = 0.05). Wisconsin clinicians were oversampled in 2002 to facilitate a within-state analysis of the impact of a television advertising campaign (not reported here). The probability of the same clinician being sampled in both 1999 and 2002 was low, and the samples were considered independent in the analyses.

### Questionnaire

The preintervention questionnaire was mailed to Wisconsin clinicians in April 1999 and to Minnesota clinicians in November 1999. During March–May 2002, the postintervention questionnaire was mailed to clinicians in both states. The questionnaires contained a cover letter explaining the purpose of the survey; 2 follow-up reminders were sent to maximize compliance. The preintervention and postintervention questionnaires were identical in their measures of knowledge, beliefs, and decision making and differed only in the addition of questions to the preintervention questionnaire regarding clinician opinion for effective campaign materials (for planning purposes) and the addition of questions to Wisconsin's postintervention questionnaire about the television advertisements.

**Figure 1:**
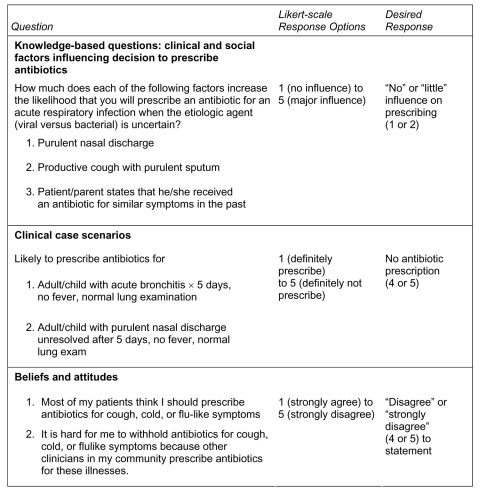
Representation of survey items assessed in 1999 and 2002 among Wisconsin and Minnesota clinicians.

After determining practice setting and basic demographics, clinicians caring for adults were asked to estimate the proportion of adult patients who requested antimicrobial agents for cough, cold, or flulike symptoms. Likewise, those caring for children were asked to estimate the proportion of parents who requested antimicrobial agents for their child. The survey questionnaire (Figure 1) then asked a series of questions to assess 1) the influence of 2 nonpredictive clinical factors (i.e., clinical symptoms or signs characteristic of both viral and bacterial infections, which therefore did not necessarily warrant antimicrobial drug therapy) and 1 social factor on the decision to prescribe antimicrobial drugs; 2) the likelihood of antimicrobial agent prescribing in adult and pediatric clinical case scenarios for URIs and bronchitis; and 3) perceptions and beliefs regarding patient expectations and peer-established norms. In addition, questions regarding exposure to and perceived impact of the WARN campaign were asked on the postintervention questionnaire (Wisconsin clinicians only). For most questions, the responses were based on a 5-point Likert-scale (e.g., "strongly disagree to strongly agree"). The Likert responses were dichotomized into desired and undesired responses (Figure 1); responses were classified as "desired" if they were consistent with national pediatric and adult clinical practice guidelines or the educational goals of the WARN campaign. The influence of social factors, patient expectations, and peer-established norms on clinical decision-making was considered "undesired" since each was an inappropriate reason for antimicrobial drug prescribing.

### Statistical Analysis

For the clinician-reported estimates of the percentage of patients or parents who requested an antimicrobial drug, we compared the distribution of responses for each year and state. We used the 1-sided Jonckheere-Terpstra test, a generalization of the nonparametric Mann-Whitney U test, to compare within-state distributions for 1999 and 2002. The null hypothesis was that the distributions did not differ between these 2 periods.

For each Likert-scale question, we calculated a ratio based on the proportion of clinicians with a desired response in 2002 (numerator) divided by the proportion with a desired response in 1999 (denominator). A ratio >1.0 indicated improvement in 2002 versus 1999. After calculating this ratio for each state, we compared the ratios between Wisconsin and Minnesota. All ratios were adjusted for clinician sex, years in practice, practice setting, and clinician type. All adjusted ratios and corresponding statistical test results were obtained directly from multivariable models similar to logistic regression models but with a log (rather than logit) link function (24). Such models permit comparison of proportions rather than odds. The models included terms for state, year, their interaction, and the control variables (clinician sex, years in practice, practice setting, and clinician type). Examining appropriate combinations of the estimated parameters from these models permitted within-year comparisons of Wisconsin versus Minnesota (e.g., baseline comparisons), within-state comparisons of 2002 versus 1999, and between-state comparisons of within-state ratios (i.e., comparisons of the 2002/1999 ratios for Wisconsin to those in Minnesota for estimating the effect in Wisconsin beyond that observed in Minnesota).

Ten percent of questionnaire responses were entered in duplicate for quality assurance. Statistical analyses were performed by using SAS version 8.2 (SAS Institute Inc., Cary, NC, USA) and EpiInfo 6 (CDC, Atlanta, GA, USA).

## Results

The survey response rates ranged from 65% to 71%. Most respondents were physicians (Table 1). The most common practice specialty was family practice. The sex distribution and years in practice in each group did not differ by state or year of survey.

**Table 1:** Response rate and respondent characteristics, Wisconsin and Minnesota clinicians, 1999 and 2002*

Characteristic	Wisconsin		Minnesota	
	1999	2002	1999	2002
Response rate (%)	71	65	69	70
Practice setting (%)				
ER/urgent care	11	22	13	14
Family practice	46	42	49	48
Pediatrics	17	13	12	16
Internal medicine	18	16	17	14
Other	8	7	9	8
Physician (%)	73	72	74	78
Male (%)	61	51	55	53
Mean y in practice	12.6	12.7	13.2	13.8

*ER, emergency room.

### Baseline Survey

**Figure 2:**
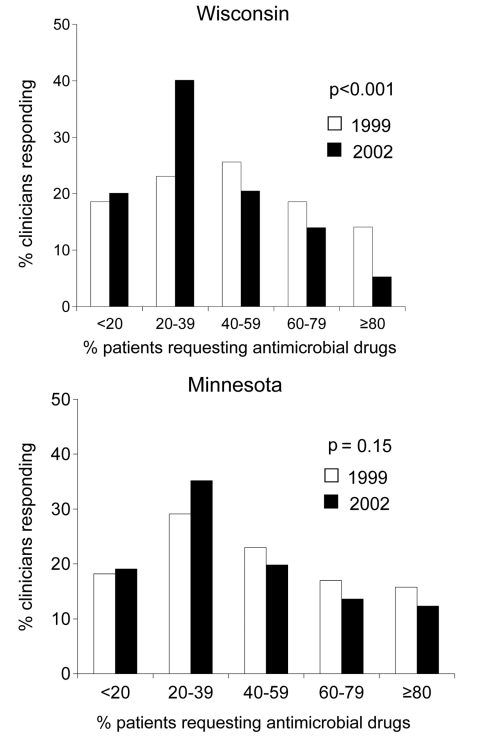
Proportion of clinicians reporting various estimates of the percentage of their adult patients who requested an antimicrobial agent for cough, cold, or flulike symptoms in 1999 and 2002.

**Figure 3:**
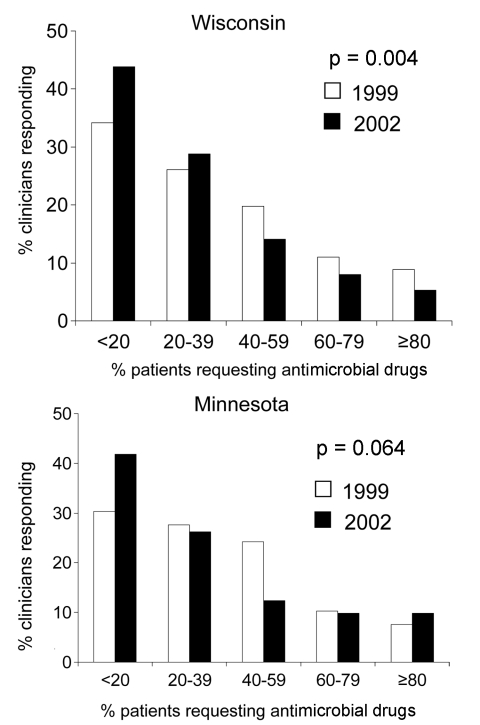
Proportion of clinicians reporting various estimates of the percentage of parents of their pediatric patients who requested antimicrobial drugs for their child's cough, cold, or flulike symptoms in 1999 and 2002.

Baseline responses were compared for Wisconsin and Minnesota in 1999, before initiation of the WARN campaign in Wisconsin. Clinicians in Wisconsin and Minnesota perceived similar proportions of their adult patients requesting antimicrobial agents (p = 0.217) (Figure 2). The median percentage of patients perceived to request antimicrobial agents was 50% in Wisconsin and 40% in Minnesota; this difference was not significant. The perceived demand by parents for antimicrobial agents to treat their child's respiratory illness was also similar between the 2 states (p = 0.473) (Figure 3); the median reported percentage of parents requesting antimicrobial agents was 25% in both states.

Clinicians in Wisconsin and Minnesota gave similar baseline responses regarding the influence of a social factor (e.g., patient states antimicrobial agents given for similar symptoms in the past) and a nonpredictive clinical factor (e.g., purulent nasal discharge) (Table 2, baseline p values not presented). However, Minnesota clinicians were significantly more likely to report that productive cough with purulent sputum would not influence their decision to prescribe an antimicrobial agent (Wisconsin 14%, Minnesota 20%, p = 0.027). For the bronchitis and viral URI case scenarios, the overall proportion of clinicians who would withhold antimicrobial agents was similar in each state. The proportion who would withhold antimicrobial agents was greater for the pediatric case scenarios than for the adult case scenarios (Table 3). Responses to the belief questions regarding patient expectations and clinician peer norms were similar between the states during the baseline period (Table 4).

**Table 2:** Influence of 2 nonpredictive clinical factors and 1 social factor on antimicrobial agent prescribing, 2002 versus 1999

Response	Proportion giving desired response (%)*					
	Wisconsin (WI)		Minnesota (MN)			
	1999	2002	1999	2002	WI % 2002/WI % 1999 (adjusted)†‡	MN % 2002/MN % 1999 (adjusted)†‡
Purulent nasal discharge	34	61	42	54	1.71 (p<0.001)	1.24 (p = 0.054)
Productive cough with purulent sputum	14	36	20	31	2.61 (p<0.001)	1.31
Received antimicrobial agents for similar symptoms in past	57	72	63	70	1.20 (p = 0.015)	1.10

*Desired response: presence of the factor had little or no influence on the decision to prescribe. †Only significant p values presented; p values for baseline comparisons not presented. ‡Ratios and corresponding p values adjusted for sex, years in practice, practice setting, and clinician type.

**Table 3:** Responses to clinical case scenarios for viral upper respiratory infection and bronchitis, 2002 versus 1999

Response	Proportion giving desired responses for both scenarios (%)*					
	Wisconsin (WI)		Minnesota (MN)			
	1999	2002	1999	2002	WI% 2002/WI%1999 (adjusted)†‡	MN% 2002/MN% 1999 (adjusted)†‡
Adult case scenarios	43	64	46	59	1.45 (p = 0.001)	1.28 (p = 0.023)
Pediatric case scenarios	62	74	66	68	1.16 (p = 0.058)	0.98

*Desired response to all scenarios—not prescribe or definitely not prescribe. †Only significant p values presented; p values for baseline comparisons not presented. ‡Ratios and corresponding p values adjusted for sex, years in practice, practice setting, and clinician type.

**Table 4:** Perceptions and beliefs regarding patient expectations and peer-established norms, 2002 versus 1999

Belief or attitude*	Proportion giving desired response (%)†					
	Wisconsin (WI)		Minnesota (MN)			
	1999	2002	1999	2002	WI % 2002/WI % 1999 (adjusted)‡§	MN % 2002/MN % in 1999 (adjusted)‡§
Most of my patients think I should prescribe for cough, cold, or flulike symptoms.	36	42	42	48	1.19	1.07
It is hard for me to withhold antibiotics because other clinicians in my community prescribe them for cough, cold, or flulike illness.	62	71	64	64	1.18 (p = 0.014)	1.00

*See Figure 1 for complete text of each statement. †Desired response—disagree or strongly disagree. ‡Only significant p values presented; p values for baseline comparisons not presented. §Ratios and corresponding p values adjusted for sex, years in practice, practice setting, and clinician type.

We also compared baseline responses between states for clinicians in practice ≤10 years and those in practice >10 years. For those in practice ≤10 years, a higher proportion of Minnesota clinicians indicated that their decision to use antimicrobial agents was not influenced by purulent nasal discharge (Wisconsin 36%, Minnesota 51%, p = 0.024) or cough with productive sputum (Wisconsin 12%, Minnesota 25%, p = 0.005). Responses to the influence of the social factor and responses to the adult and pediatric case scenarios were similar between the states. For clinicians in practice >10 years, responses were similar between the 2 states regarding the influence of nonpredictive clinical factors and the social factor. Compared to Wisconsin clinicians practicing >10 years, a higher proportion of Minnesota clinicians in long-term practice indicated they would withhold antimicrobial agents in the pediatric case scenarios (p = 0.048) and the adult case scenarios (p = 0.118).

Within Wisconsin, some baseline responses differed according to length of time in practice. A significantly higher proportion of clinicians practicing ≤10 years gave the desired responses for the pediatric (p = 0.027) and adult (p = 0.002) case scenarios. They were also more likely to give the desired response regarding the influence of a social factor (i.e., patient states antimicrobial agents were given for similar symptoms in the past) (p = 0.043). Wisconsin clinicians in practice ≤10 years and those in practice >10 years gave similar responses regarding the influence of the nonpredictive clinical factors (i.e., purulent nasal discharge and productive cough).

When specialties were compared, a higher proportion of pediatric clinicians gave desired responses than clinicians in other specialties on most outcome measure in both Wisconsin and Minnesota. Baseline comparisons between physicians and nonphysicians did not show a consistent tendency for 1 group to perform better than the other.

### Follow-up Survey

In 2002, Wisconsin clinicians perceived less demand for antimicrobial agents among adult patients compared with 1999 (p<0.001) (Figure 2). Based on clinician estimates, the median percentage of patients who requested an antimicrobial agent for cough, cold, or flu symptoms decreased from 50% in 1999 to 30% in 2002. Minnesota clinicians also perceived a decrease in the percentage of patients who requested antimicrobial agents, but the difference was not significant (p = 0.152) (Figure 2); the median percentage of Minnesota patients requesting antimicrobial agents decreased from 40% in 1999 to 30% in 2002.

In both states, a decline was noted in the perceived parental demand for antimicrobial agents to treat pediatric respiratory illness (Figure 3). The temporal change was significant in Wisconsin (p = 0.004) and approaching significance in Minnesota (p = 0.064). The median reported percentage of parents who requested an antimicrobial agent decreased from 25% in 1999 to 20% in 2002 in both states, but the distribution around the medians differed significantly between the states.

In Wisconsin, significant improvement occurred in the responses to the 2 questions about nonpredictive clinical factors and the social factor that may increase the likelihood of prescribing antimicrobial agents (i.e., purulent nasal discharge, productive cough, and patient or parent statement that antimicrobial agents were prescribed for similar symptoms in the past) (Table 2). Wisconsin clinicians were significantly more likely to report that each factor did not influence antimicrobial agent prescribing practices in 2002 compared with 1999. In Minnesota, a significant improvement occurred in responses regarding the influence of purulent nasal discharge, but no significant change occurred for the other 2 factors. Overall, Wisconsin clinicians demonstrated significant improvement regarding the influence of purulent nasal discharge (p = 0.044) and productive cough (p = 0.010) after accounting for temporal changes in Minnesota.

Both Minnesota and Wisconsin clinicians improved in their responses to the adult case scenarios for URI and bronchitis (Table 3). The magnitude of improvement was greater for Wisconsin clinicians, but the improvement in Wisconsin was not significant after accounting for the secular trend in Minnesota. In the pediatric case scenarios, Wisconsin clinicians improved from 1999 to 2002 (p = 0.058), while the responses of Minnesota clinicians were essentially unchanged (p = 0.807).

Wisconsin clinicians demonstrated a modest improvement from 1999 to 2002 in response to questions concerning perceived clinician peer norms and patient expectations, but the changes in Wisconsin were not significant after accounting for temporal changes in Minnesota (p = 0.103 and 0.519, respectively, Table 4).

### Subgroup Analysis

Responses were analyzed separately for clinicians who had practiced >10 years (1999, n = 198; 2002, n = 243) and those in practice ≤10 years (1999, n = 187; 2002, n = 239). In Wisconsin, clinicians who were in practice for the longer period demonstrated significant improvements regarding the likelihood of prescribing antimicrobial agents for purulent nasal discharge (2002 to 1999 ratio = 1.61, p = 0.005) and productive cough (2002 to 1999 ratio = 2.35, p = 0.001). They also improved in their responses to the influence of patient/parent statement that antimicrobial agents were prescribed for similar symptoms in the past (2002 to 1999 ratio = 1.36, p = 0.012). Wisconsin clinicians practicing >10 years also demonstrated significant improvements in the adult case scenarios (2002 to 1999 ratio = 2.00, p<0.001) and the pediatric case scenarios (2002 to 1999 ratio = 1.43, p = 0.002)] and in the questions concerning patient expectations and peer norms. The 2002 to 1999 ratio was 1.40 (p = 0.031) for patient expectations and 1.28 (p = 0.021) for peer norms. However, only the responses to the pediatric case scenarios improved significantly among physicians in practice >10 years (p = 0.027) after accounting for the secular trend in Minnesota.

Wisconsin clinicians in practice ≤10 years improved in fewer areas. They improved significantly in responses regarding the influence of purulent nasal discharge (p<0.001) and productive cough (p<0.001) on antimicrobial agent prescribing practices, and both factors remained significant after accounting for the secular trend. No significant change occurred in the other responses. A direct comparison between Wisconsin clinicians practicing ≤10 years to those practicing >10 years demonstrated no significant difference with regard to improvement in knowledge or the response to the clinical scenarios.

### Familiarity with WARN and WARN Materials

Ninety percent of primary care clinicians in Wisconsin had heard of WARN. Of those, 70% had used WARN patient education materials. Of those using the materials, 41% reported that they were "very useful," and 59% said that they were "somewhat useful."

## Discussion

The results of this study demonstrated significant improvement among primary care clinicians in multiple outcome measures after a multifaceted educational campaign to promote appropriate antimicrobial drug use in Wisconsin was implemented. From 1999 to 2002, clinicians perceived less patient or parent demand for antimicrobial agents and were less likely to report that antimicrobial agent prescribing was influenced by social and nonpredictive clinical factors. Clinicians demonstrated improved decision-making in adult and pediatric case scenarios for URIs and bronchitis and perceived less pressure from patients and peers to prescribe. Minnesota clinicians also demonstrated improvement in some of these factors, but the magnitude of improvement was consistently greater among Wisconsin clinicians, and the improvements in several of these factors in Wisconsin remained significant even after the secular trend in Minnesota was accounted for.

The greater improvements in responses from Wisconsin clinicians over time compared with Minnesota clinicians suggest that the WARN program had a positive effect on clinician knowledge and beliefs. This effect is supported by the observations that among Wisconsin clinicians, a high level of recognition and acceptance of WARN was achieved, and that from 2000 through 2002, the use of WARN educational materials was widespread.

The WARN campaign was initiated in 1999 as a large-scale demonstration project designed to promote appropriate antimicrobial agent use for outpatient respiratory illness. It was the largest of its kind in the United States and the first to evaluate whether prescribing practices could be improved for an entire state. At the time the project was initiated, clinicians perceived a high demand for antimicrobial agents and displayed prominent gaps in knowledge regarding outpatient antimicrobial agent use for URIs and bronchitis (4,25–28). At the same time, knowledge of appropriate antimicrobial agent use was limited among much of the general public (27–30).

The results of this study are consistent with those of other studies demonstrating the impact of multifaceted educational efforts that specifically focused on physicians, patients, or the general public. Campaigns that focused on parents, using videotaped presentations in pediatric waiting rooms, showed modest to significant improvement in parental knowledge and attitudes about appropriate antimicrobial agent use (31,32), but in-service reviews of judicious antimicrobial agent use guidelines for clinicians had no effect on antimicrobial agent prescribing rates (32). Intensive education of both the clinicians and the community has led to significant decreases in antimicrobial agent prescription rates, as shown by studies in Knox County, Tennessee (33), eastern Massachusetts, northwest Washington state (34), the Denver metropolitan area (35), northern Wisconsin communities (36), and rural Alaskan villages (37). Only the Tennessee study addressed a large general population, whereas the other studies focused on rural communities or managed care populations.

In subgroup analysis, we found the greatest improvements among Wisconsin clinicians who had been in practice for >10 years. Although these improvements coincided with the secular trend observed in Minnesota, they demonstrate that this group of physicians should be targeted for further education. One potential explanation for why clinicians who were trained more recently showed fewer improvements is that they might already have a greater awareness of issues regarding increasing antimicrobial drug resistance and were trained more rigorously in the principles of judicious antimicrobial drug use. This hypothesis was supported by the baseline assessment, which showed that clinicians practicing ≤10 years performed better on the clinical case scenarios. These results parallel other findings that clinicians who are temporally further away from medical training programs prescribe antimicrobial drugs more frequently (38), although this finding has not been consistently demonstrated (4,26).

This study did not include objective measures of antimicrobial drug prescribing. Prior studies have shown that changes in knowledge and attitudes do not necessarily translate into changed in clinical practice (4,25). The medical culture surrounding antimicrobial drug prescribing in the United States is influenced by multiple external factors (e.g., peer practices, pharmaceutical detailing, geographic region, and managed care restrictions), and these may influence practice more than knowledge of current guidelines. The accompanying study by Belongia et al. addresses the impact of WARN on antimicrobial drug prescribing rates in Wisconsin relative to those of Minnesota (23).

A limitation of this study was the lack of statistical power to detect modest improvements after accounting for the secular trend in Minnesota. The magnitude of improvement in Wisconsin consistently exceeded that in Minnesota, but the difference was often deemed statistically insignificant. A larger sample size may have provided additional power to distinguish between these smaller differences. In addition, a higher proportion of Minnesota clinicians gave the correct or desired response to several of the baseline survey items compared with Wisconsin clinicians, although many of these differences were not statistically significant. Minnesota clinicians may have had less room to improve and might have already been more familiar with recommendations regarding judicious antimicrobial durg use; therefore, Minnesota might not have been wholly optimal as a control state. Additionally, Minnesota clinicians and public along the Wisconsin-Minnesota border may have been exposed to WARN materials and advertisements. An added limitation is that this study included only 2 states. If substantially more resources had been available, a controlled, multistate intervention study would have provided more robust and generalizable results. A larger study may no longer be feasible, given the success of current campaigns in promoting awareness on a national level. The efficacy of large multifaceted interventional campaigns will be difficult to evaluate because unexposed populations no longer exist.

In conclusion, this study suggests that the WARN campaign had at least a modest positive effect on the knowledge and decision-making of primary care clinicians in Wisconsin. Clinicians in practice >10 years demonstrated the greatest improvements and may benefit most from educational interventions. Further research should include the development and evaluation of interventions to improve antimicrobial agent selection (narrow-spectrum versus broad-spectrum) and an assessment of new clinical strategies to optimize antimicrobial agent usage (e.g., a 72-hour waiting period for selected patients with mild acute otitis media) (39,40). The documented success of these smaller campaigns in changing the medical culture surrounding antimicrobial drug prescribing has prompted its expansion to the national level. A national public education campaign was launched by CDC in September 2003 to further generate provider and public awareness of these issues and to curb the inappropriate use of antimicrobial drugs.
